# Microwave-Assisted Hydrodistillation of the Insecticidal Essential Oil from *Carlina acaulis*: A Fractional Factorial Design Optimization Study

**DOI:** 10.3390/plants12030622

**Published:** 2023-01-31

**Authors:** Eleonora Spinozzi, Marta Ferrati, Desiree Lo Giudice, Eugenio Felicioni, Riccardo Petrelli, Giovanni Benelli, Filippo Maggi, Marco Cespi

**Affiliations:** 1 Chemistry Interdisciplinary Project (ChIP) Research Center, School of Pharmacy, University of Camerino, via Madonna delle Carceri 9/B, 62032 Camerino, Italy; 2 Department of Agriculture, Food and Environment, University of Pisa, via del Borghetto 80, 856124 Pisa, Italy

**Keywords:** Asteraceae, bioinsecticide, carlina oxide, ETHOS X, GC-MS, green pesticide

## Abstract

Recently, microwave-assisted hydrodistillation (MAH) has been reported as an innovative technique leading to increased essential oil (EO) extraction yield, coupled with reduced extraction time and energy costs. The EO of *Carlina acaulis* L. (Asteraceae), mainly constituted by carlina oxide (>95%) and conventionally obtained through traditional hydrodistillation (HD), has been reported as extremely effective against several arthropod vectors and pests of medical and economic importance with limited impact on non-target species, including mammals. This study aimed to the optimization of the EO extraction through MAH by using a one-step design of experiments (DoE) approach that allowed us to relate the characteristics of the produced EOs with the applied experimental conditions using mathematical models. The preliminary screening allowed us to optimize the protocol only by the extraction time, skipping complex data analysis. Moreover, the comparison of the optimized MAH conditions with traditional HD pointed out the higher efficiency of MAH in terms of EO yield (0.65 and 0.49% for MAH and HD, respectively) and extraction time (210 min for MAH). The results obtained confirmed the promising role that MAH could have in *C. acaulis* EO extraction, with increased yield and reduced extraction time, water consumption, and energy costs, and being employable on an industrial scale, with special reference to insecticidal and acaricidal formulations.

## 1. Introduction

Botanical insecticides based on essential oils (EOs) or their main components have recently attracted the attention of agrochemical companies as valuable tools for integrated pest management (IPM). EOs represent a sort of ‘highly concentrated products’ generally more effective on insects and mites than many other kinds of botanicals [1]. Indeed, entomological research pointed out that EOs are often characterized by significant insecticidal and acaricidal efficacy, limited impact on non-target organisms, and synergistic multitarget effects of their main components, thus there is a scarce possibility to cause resistance in targeted pests [2,3,4,5].

To satisfy the requirements of the agrochemical industry for industrially scalable processes, EOs should be obtained in high yields from available plant biomasses, possibly cultivable on a large scale. For this reason, in the last years, several advanced extraction techniques capable of boosting the EO extraction yield have been introduced. One of them is microwave-assisted hydrodistillation (MAH), which is a green and eco-friendly technique largely employed in the extraction of EOs from natural sources [6]. The extraction principle relies on the absorption of microwave electromagnetic energy by polar molecules (e.g., water) inside the plant material through two mechanisms: dipole rotation and ionic conductance. These processes cause an increase of pressure inside the cells leading to the disruption of membranes and the release of volatile bioactive compounds, which evaporate and are subsequently recovered through a condensation system. When compared with traditional extractive systems, such as hydrodistillation (HD) or steam distillation (SD), MAH showed significantly higher recovery of EOs along with shorter extraction times and water and energy savings [7]. Indeed, if compared with convection and conduction systems, MAH ensures more rapid heating exploiting the water inside the plant matrix without the need for additional water. In modern MAH systems, the oven frequency is set at 2.45 GHz while the microwave power is digitally controlled allowing the setting of the optimum extraction conditions [8,9].

In this work, a MAH optimization study for the obtaining of the *Carlina acaulis* L. (Asteraceae) root EO was performed. This EO has been reported as a promising candidate ingredient for biopesticide formulations given its high efficacy against several arthropod pests and vectors with an impact on agriculture and human health [10,11,12,13,14]. In addition, this product is scarcely toxic to non-target organisms [15], which is a hallmark strictly required by the European Union for registration as botanical insecticide or acaricide. The active ingredient of *C. acaulis* EO is the polyacetylene carlina oxide, which occurs in percentages higher than 90% [16].

The aim of the presented work was to analyze the most influential parameters of MAH of *C. acaulis* EO to clarify their effects on the extraction process and ultimately optimize it in terms of EO yield and carlina oxide content. To do this, the design of experiments (DoE) represents a rather convenient set of methodologies since it allows to rationalize and reduce the number of experimental procedures, obtaining sufficient data for a complete understanding of the whole process. In general, the parameters potentially influencing the performance of MAH are microwave power, extraction time, water/root ratio, preliminary soaking, prior milling, and extraction cycles. While the first three parameters are commonly studied for the process, the last three are poorly evaluated and there are few data supporting or contradicting their usefulness in the context of MAH technology. So, this study aimed to optimize the EO extraction through MAH by a one-step DoE approach and to compare the evaluation of this advanced technique with traditional HD.

## 2. Results

### 2.1. Preliminary Screening

**Figure 1:**
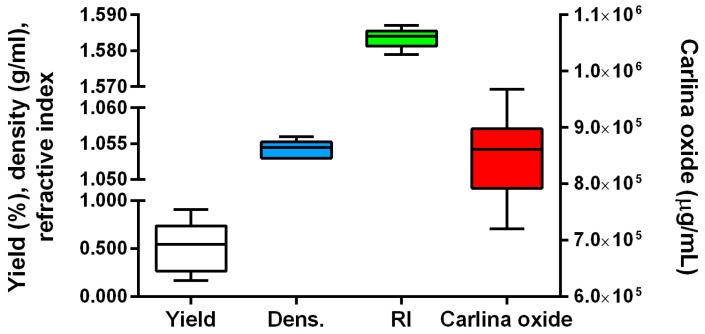
Box plots displaying values distribution of yield (%), density, refractive index (RI), and carlina oxide content (µg/mL). The horizontal line within the box is the median value, while the maximum and minimum values recorded are represented by the whiskers.

The DoE approach is an efficient tool used for identifying the connections existing between causes (factors or variables) and responses (measured properties of the EO in this case) and is employed for factor screening, factor analysis, and process optimization. This approach was chosen for the optimization of MAH of the *C. acaulis* root EO. The aim of the screening phase was the identification of the significant parameters affecting the quantity and quality of EO. Milling (Mi) and moistening (Mo) processes, and the efficiency of fractional runs (Cycles) were taken into consideration. Commonly investigated factors such as extraction time (ET), microwave power (MP), and the water-to-matrix ratio (W) were also evaluated. Data distributions of all responses (i.e., EO yield, density, refractive index, and content of carlina oxide) determined for the EOs (16 experimental runs of the screening design) were preliminarily analyzed using box plots (Figure 1).

**Figure 2:**
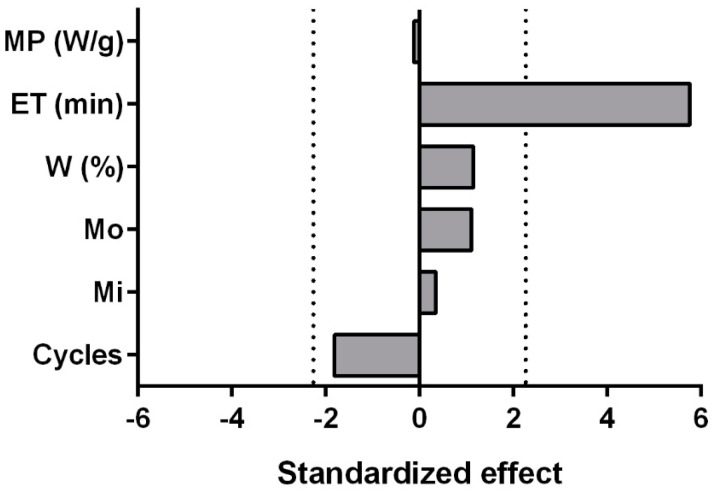
Pareto plots showing the factors that influenced the essential oil (EO) yield as determined in the fractional factorial design. The dot lines represent the statistically significant limits (*t*-value_α/2_ of a t-distribution with degrees of freedom equal to the degrees of freedom for the error term) when the variables are reported in terms of standardized effect (*t*-value of the coefficient). MP is the microwave power; ET is the extraction time; W is the percentage of water added to the seeds; Mo is the moistening process; Mi is the milling process.

The measured refractive index (1.585 and 1.584 for MAH and HD EOs, respectively) and density (1.056 and 1.054 g cm^−3^ for MAH and HD EOs, respectively) values were comparable for all 16 EOs, with differences at the third decimal digit, which was the lowest sensitivity limit of the instrument employed. For this reason, these responses were not considered reliable for discrimination between EOs obtained from different runs and they have not been further evaluated in this work. Conversely, the EO yield and carlina oxide content changed significantly between the 16 experiments, ranging from 0.17 to 0.91% (*w/w*), and from 72 to 97%, respectively. Therefore, these responses were the only two considered for fractional factorial design (FFD) analysis. The regression analysis (the detailed results of the regression analysis are reported in Section S1 in Supplementary Materials) demonstrated that the content of carlina oxide could not be adequately described by the used model since no significant regression was determined. Probably, the carlina oxide content variability (Figure 1) was linked to the intrinsic variability of the samples or depended on other factors that were not investigated in the study and that are still unknown. However, since the FFD design aims only to screen the factors and not to define a predictive model, the only relevant consideration is that the studied factors did not affect the amount of carlina oxide in the EO. The model fitting for the EO yield results was statistically significant and the adjusted determination coefficient (R^2^_adj_) was 0.69, while the residuals analysis did not show issues or anomalies (Section S1 in Supplementary Materials). In these conditions, the regression model was considered reliable, at least for a screening. From the model coefficient analysis, it was underlined that the only significant parameter was the ET, displaying a positive correlation with the EO yield. The other analyzed factors had no significant effect on the extraction yield, as reported in the Pareto plots (Figure 2).

**Figure 3:**
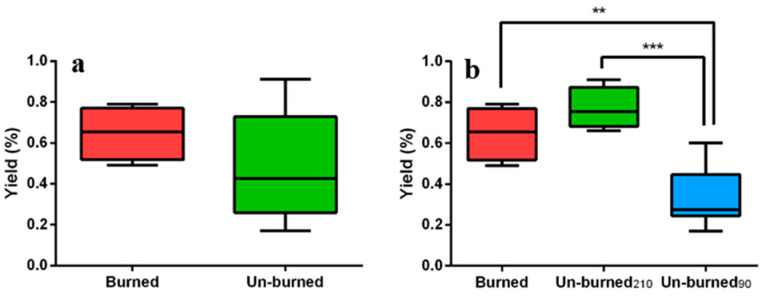
Box plots showing the yield values for burned and un-burned samples (**a**) and for burned vs. un-burned samples subject to microwave irradiation for 90 or 210 min (**b**). The two groups in panel A were compared with an unpaired *t*-test, while the three groups of panel B were compared with ANOVA followed by Tukey’s HSD test. The significance was reported in terms of the *p*-value as follows: no asterisks for *p* > 0.05; * for 0.05 < *p* < 0.01; for ** 0.01 < *p* < 0.001; *** for *p* < 0.001. The horizontal line within the box is the median value, while the whiskers represent the maximum and minimum values recorded; the top of the rectangle indicates the third quartile, while the bottom of the rectangle indicates the first quartile.

**Figure 4:**
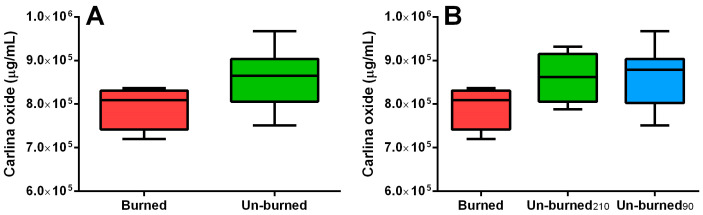
Box plots showing carlina oxide content value distributions for burned and un-burned samples (**A**) and for burned vs. un-burned samples subject to microwave irradiation for 90 or 210 min (**B**). The two groups in panel A were compared with an unpaired *t*-test, while the three groups in panel B were compared with ANOVA. The significance was reported in terms of the *p*-value as follows: no asterisks for *p* > 0.05; * for 0.05 < *p* < 0.01; for ** 0.01 < *p* < 0.001; *** for *p* < 0.001. The horizontal line within the box is the median value, while the whiskers represent the maximum and minimum values recorded; the top of the rectangle indicates the third quartile, while the bottom of the rectangle indicates the first quartile.

It was also interesting to investigate the sample conditions at the end of the extraction. In detail, samples 3, 4, 11, and 12 (Table 1 reported in Section 4.4) were burned or partially burned. All these samples shared two common experimental conditions: a long extraction time (210 min) and a low water amount (65%). None of the un-burned samples were processed by applying the same values of ET and W at the same time. The sample burning did not represent an independent variable and cannot be directly considered during regression analysis. For these reasons, the eventual effect of burning on EO yield and carlina oxide content has been evaluated using the *t*-test or ANOVA. The results of hypothesis tests (Figure 3a and Figure 4A) clearly showed that EO yield and carlina oxide content were not influenced by the sample burning. Moreover, the un-burned samples were divided into two different groups according to the ET (Figure 3b and Figure 4B) since the latter represented the only relevant variable for the yield. This comparison led to the conclusion that a different ET causes a variation in EO yield but not in the carlina oxide content independently on sample burning, confirming the relevance of the FFD results. However, the exact moment of burning, which was estimated to be between 90 and 210 min, is unknown.

### 2.2. The Effect of the Extraction Time

**Figure 5:**
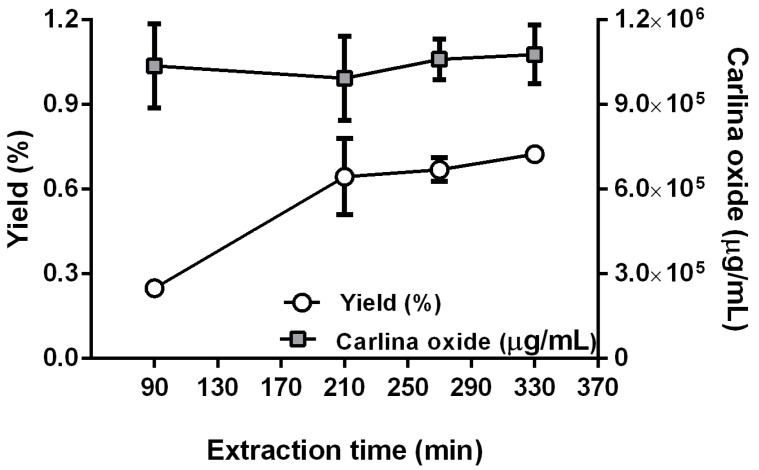
Influence of the extraction time on essential oil (EO) yield (%) and carlina oxide content (µg/mL) for the extraction runs performed after the fractional factorial design (FFD) (Section S1 of the Supplementary Materials). Results of different extraction time points for both responses were matched with ANOVA and further analyzed using Tukey’s HSD test for significant differences. Different letters show significant differences (Tukey’s HSD test, *p* < 0.05).

In the preliminary screening of this work, it was reliably demonstrated that the ET was the only factor affecting the yield of the EO. Consequently, to deeply understand the influence of this parameter on the process, further experimental runs were performed varying the ET from 90 to 330 min and setting constant the un-relevant factors (Mo, Mi, MP, W, and cycles). Mo, Mi, and cycles were not performed (levels not applied, NA, in Table S2, Supplementary Materials) while W and MP were set to prevent samples burning (85% and 1 W/g, respectively, Table S2, Supplementary Materials). The new run conditions are detailed in Table S2 of the Supplementary Materials, while the yield and carlina oxide concentration obtained for each extraction are reported in Figure 5.

The extraction time did not affect the carlina oxide content that remained constant in each EO (ANOVA analysis did not reveal significant differences), corroborating the results of the screening also for longer ET. Regarding the EO yield, it was higher for the extraction carried out for 210 min (Tukey’s HSD test evidenced statistically significant differences between 90 and 210 min runs), remaining almost constant for longer times (the EO yields at 210, 270, and 330 min were not statistically different, while the values at 90 min were statistically different with respect to the three EO yield values, according to Tukey’s HSD test). The plant material was not burned for any extraction reported in Figure 5. Moreover, to confirm that no more EOs could be extracted, the last run was re-started after 330 min and no additional EO was produced. These results demonstrated that 210 min represents the ideal extraction time to obtain the EO from *C. acaulis* root using MAH.

### 2.3. Comparison of MAH and HD

**Figure 6:**
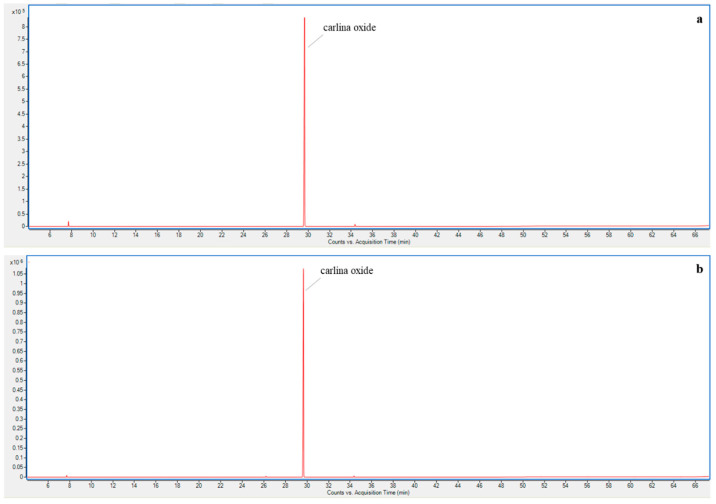
GC-MS chromatograms of HD (**a**) and MAH (**b**) *Carlina acaulis* essential oils.

To compare the EO yield obtained through MAH, a conventional HD run was carried out setting the same conditions of the optimized MAH extraction procedure. In particular, 85–15% and 210 min were maintained for the water-to-matrix ratio (W) and ET, respectively. The yields obtained were 0.49 and 0.65% for HD and MAH, respectively. The carlina oxide content resulting from the quantitative GC analysis was not significantly different between the two EOs. Additionally, both the EOs were analyzed qualitatively through GC-MS and no significant differences in the chemical compositions were detected. In fact, carlina oxide was the main constituent, representing 97.9% and 98.6% of the total composition for HD and MAH EOs, respectively, followed by benzaldehyde (1.4% and 0.6%, respectively), and *ar*-curcumene (0.1% and 0.3%, respectively). Figure 6 reports the chromatograms obtained from the GC-MS qualitative analyses, for which 99.3 and 99.5% of the total peak areas were identified for HD and MAH EOs, respectively.

## 3. Discussion

Prior to the preliminary screening, the different measured responses were evaluated in terms of their ability to discriminate between the diverse extraction conditions. Refractive index (RI) and density values were comparable for all the obtained EOs and were not used as quality indicators of the samples. This result was not surprising, since those parameters can be employed to discriminate EOs only if such EOs contain different compounds characterized by diverse RI and density values, as can be deduced from studies previously reported [17,18]. Similar results have been previously reported for the study of the influence of different MAH operating conditions on the EO compositions of ajowan (*Trachyspermum ammi* (L.) Sprague) and hemp (*Cannabis sativa* L.) [8,9]. These studies were conducted with a different DoE design containing repeated runs and consequently, the poor fitting could be attributed to the sample’s intrinsic variability rather than unknown factors. However, in this work, RI and density values strongly suggest that the content of carlina oxide in the *C. acaulis* EOs was almost constant, independently on the experimental conditions applied, and the observed differences were exclusively related to the sample intrinsic variability.

The extraction yield was affected only by the ET, while the other factors did not show any significant effect. This result is partially unexpected. In fact, the study by Mazzara et al. [18] demonstrated that W, Mo, Mi, and cycles did not improve the EO yield of *T. ammi* during MAH, demonstrating the scarce relevance of these parameters on fibrous samples. On the contrary, the MP result was rather surprising considering the literature findings [8,19] regarding other plant matrices. To our knowledge, the *C. acaulis* root is the first plant matrix in which MP did not play any role in the EO extraction, at least for the conditions investigated in this study. In this context, worthy of notice is that the MP applied felt in the range of medium-high, comparable with many works published on MAH extraction; in addition, higher values should not be applied to avoid the risk of flaming (the extreme conditions of MP and ET applied in this work represent the limit since they determined the burning of the residual matrix).

A screening design is usually followed by a more appropriate DoE to deeply understand the factors’ effects and to build a predictive model which allow process optimization; this strategy is of fundamental importance to maximize the cost/benefit ratio of the full project [20]. However, in this study, the preliminary screening reliably demonstrated that the ET was the exclusive factor affecting the process, avoiding further DoE studies, as was initially planned. The results described in Section 2.2 revealed that the ideal extraction time to obtain the EO from *C. acaulis* root using MAH is 210 min. Moreover, the yield of the EO obtained from MAH resulted 0.65% and this was higher than that obtained by HD used as a comparison (0.49%). These results confirm the efficiency of the conditions described above and demonstrate that the protocol for *C. acaulis* EO MAH extraction was optimized, being employable on an industrial level. Regarding the chemical compositions of the EOs found in this study, they were comparable with that reported in the literature and obtained by HD [10,12].

To the best of our knowledge, no studies on the EO extraction from *C. acaulis* using MAH are available, making our study the first to be undertaken. Consequently, a comparison of the results obtained with other works is not possible. However, studies on the MAH of many plants have been reported in the literature, using a similar DoE approach, to which it is possible to compare the parameters evaluated and their impact on EO yield and chemical composition. Mazzara et al. [18] used a two-step DoE strategy to minimize the number of tests and increase the cost/benefit ratio in a comparable study using ajowan seeds. A correlation between the three major compounds, namely thymol, *p*-cymene, and *γ*-terpinene, and the experimental conditions were detected and evaluated. Their levels resulted modified as time and power values changed. For example, the most active ingredient, thymol, rose in concentration as MP increased and within a short period of time, exhibiting an inverse correlation with respect to *p*-cymene and *γ*-terpinene. Fiorini et al. [8] reported another study using a two-step DoE on dry inflorescences of *C. sativa*, and the results showed that MAH treatment, using high MP and relatively long ET, significantly increased the content of cannabidiol (CBD) in the EO, maintaining high EO yield values when compared to conventional HD.

## 4. Materials and Methods

### 4.1. Plant Material

The *C. acaulis* roots, consisting of dry coarsely shredded material, consequently already homogenized, were bought from A. Minardi & Figli (Bagnacavallo, Ravenna, Italy), batch No. C-210920250920, and derived from a wild population collected in Albania in 2020.

### 4.2. Sample Preparation

Plant material was processed as such, or pre-treated, via milling and/or moistening, according to the screening design experimental conditions reported below (Section 4.4 Table 1). The milling process was performed with a stainless-steel shredder from Albrigi Luigi Srl (Verona, Italy, code E0585) at a power of 1100 W, and equipped with a sieve with 1.5 mm size holes (images of coarsely shredded and powdered material are reported in Supplementary Materials Section S4). The moistening process was carried out before the extraction for 16 h, using the same amount of distilled water required for each screening experiment (Section 4.4).

### 4.3. Microwave-Assisted Extraction (MAH)

The EO samples were obtained by Milestone ETHOS X (Milestone Srl, Sorisole, Italy) microwave extraction equipment. This device is equipped with a 2.45 GHz microwave reactor outfitted with an infrared sensor that monitors the temperature and two magnetrons achieving a maximum power of 1800 W (2 × 950 W). All the tests were performed at atmospheric pressure in a glass reactor (Pyrex), with a total capacity of 5 L, closed with a glass lid. The system was equipped with a stainless-steel Clevenger-type device on the top of the oven (‘Fragrances mode’ set up) connected to a Chiller (Smart H150-2100S, Labtech Srl, Sorisole, Italy) set to hold the temperature of the condenser at 8 °C. All 16 screening runs (Section 4.4) were carried out on 1 kg of loaded sample constituted by shredded (or powdered) roots and water in variable percentages (W). The operative conditions, including microwave irradiation power (MP) and extraction time (ET), in addition to the pre-treatment milling (Mi) and moistening (Mo), were varied during the screening. Experimental runs were also performed in one step or splitting the whole process into two irradiation phases separated by the time required to reach a temperature of 50 °C in the reactor (Cycle). During the first and second steps, the weight of the loaded samples was checked and restored by adding water, if necessary. The experimental values of the variables (also defined as factors) W, MP, ET, Mi, Mo, and Cycle are reported in the next section. At the end of each experiment, the EO was obtained through the separation from the aqueous layer. The samples were stored in glass vials, which were then wrapped with aluminum foil to avoid light exposure and kept at −20 °C until further chemical analysis.

### 4.4. Design of Experiment (DoE)

The screening of the parameters that can possibly influence the MAH process was carried out using a two-level quarter FFD, described by:

$$N=l^{f-p/2}$$

where *N* represents the number of extractions to be performed (experimental runs), *f* is the number of investigated factors (6 in this study), *l* is the levels for each factor (number of experimental values that each factor can assume in the experimental runs, 2 in this study), and *p* is the partitioning of the design. The partition selected was 4; that is, the design was fractioned to a quarter and the number of experimental runs became 16 from the original 64 (full factorial design). The chosen FFD has a resolution of IV, meaning that all the main effects can be independently estimated while two-factor interactions are aliased between them. The selected resolution is appropriate for a design intended for factor screening [20].

**Table 1:** Experimental conditions both in uncoded and coded variables of the sixteen runs carried out, according to the screening design.

Run	Uncoded Variables						Coded Variables ^a^					
	MP (W/g)	ET (min)	W (%)	Mo	Mi	Cycles	MP	ET	W	Mo	Mi	Cycles
1	1	90	65	N	N	N	-	-	-	-	-	-
2	1.45	90	65	N	Y	N	+	-	-	-	+	-
3	1	210	65	N	Y	Y	-	+	-	-	+	+
4	1.45	210	65	N	N	Y	+	+	-	-	-	+
5	1	90	85	N	Y	Y	-	-	+	-	+	+
6	1.45	90	85	N	N	Y	+	-	+	-	-	+
7	1	210	85	N	N	N	-	+	+	-	-	-
8	1.45	210	85	N	Y	N	+	+	+	-	+	-
9	1	90	65	Y	N	Y	-	-	-	+	-	+
10	1.45	90	65	Y	Y	Y	+	-	-	+	+	+
11	1	210	65	Y	Y	N	-	+	-	+	+	-
12	1.45	210	65	Y	N	N	+	+	-	+	-	-
13	1	90	85	Y	Y	N	-	-	+	+	+	-
14	1.45	90	85	Y	N	N	+	-	+	+	-	-
15	1	210	85	Y	N	Y	-	+	+	+	-	+
16	1.45	210	85	Y	Y	Y	+	+	+	+	+	+

^a^ Abbreviations for coded variables: MP (microwave power); ET (extraction time); W (percentage of water added to the roots); Mo (moistening process); Mi (milling process); N (no); Y (yes).

Each of the 16 runs required by the FFD was carried out applying two different values (levels) for each of the selected variables, as reported in Table 1, both as experimental values (uncoded variables) or coded notations (coded variables). The factor W represents the % of water in 1 kg of loaded samples, while for the categorical factors (Mo, Mi, and Cycle) the uncoded values of Y or N identified if the condition has been applied or not. Each experiment was evaluated in terms of the following responses:

$$\mathrm{EO}\;\mathrm{yield}\;(\%):\;\frac{g\;of\;EO}{g\;of\;dry\;biomass}\times 100$$

EO density (g/cm^3^) (Section 4.6.1);

EO refractive index (RI) (Section 4.6.2);

EO content of carlina oxide (Section 4.6.3).

The procedure for the FFD analysis was the multilinear regression applying a liner model (suitable for a resolution IV design):

$$y=\beta_{0}+\overset{n}{\underset{i=1}{\sum }}\beta_{i}x_{i}$$

where *y*, *β*_0_, and *β_i_* are the response, the model constant, and the model coefficients corresponding to the variables *x_i_* (linear terms), respectively. Analysis of variance (ANOVA), coefficient, and residual analyses was used for checking the fitting procedure. Minitab 18 statistical software was used for the screening design and analysis.

### 4.5. Hydrodistillation (HD)

*C. acaulis* roots were subjected to HD to compare the MAH extraction to the traditional method. The extraction time chosen was the same as the MAH validation experiment (210 min). Briefly, the dry shredded roots (150 g) were put in a 2 L round flask, and a mantle system Falc MA (Falc Instruments, Treviglio, Italy) was used as a heating system. This equipment was combined with a glass Clevenger-type apparatus. At the end of the distillation, the EO was separated from the aqueous phase and stored in a glass vial wrapped with aluminum foil and kept at −20 °C until further chemical analysis.

### 4.6. Analysis of EOs Chemical-Physical Properties

#### 4.6.1. Density Determination

A digital densimeter with an oscillating U-tube (DA-100M, Mettler Toledo, Columbus, Ohio) operating at 25 °C was used to measure the density of the EOs obtained during the FFD runs.

#### 4.6.2. Refractive Index (RI)

The RI was calculated using an Abbe refractometer (NAR-1T LIQUID, Atago Co., LTD, Minato-ku, Tokyo, Japan) at 20 °C.

#### 4.6.3. GC-MS Analysis

##### Chemicals and Reagents

HPLC-grade diethyl ether, *n*-hexane, and undecane were purchased by Sigma-Aldrich, Milan, Italy. Carlina oxide employed for standard solutions preparation was previously isolated following the procedure reported by Benelli et al. [10].

##### Preparation of Standard Solutions

A stock solution containing 1000 µg/mL of carlina oxide (obtained as described in Section 4.6.3) was prepared in HPLC-grade diethyl ether and stored at −20 °C in glass vials until chemical analysis. Other standard solutions of carlina oxide were prepared to dilute the stock solution to 100, 200, 300, 400, 600, and 800 µg/mL. A stock solution containing 10,149 µg/mL of undecane was prepared in an HPLC-grade diethyl ether and stored at −20 °C in glass vials until chemical analysis. This solution was used for the dilution of 200 µg/mL.

##### EO Characterization and Quantification of Carlina Oxide

*C. acaulis* EOs were analyzed by an Agilent 8890 gas chromatograph (GC) coupled with a single quadrupole 5977B mass spectrometer (Santa Clara, CA, USA) and an autosampler PAL RTC120 (CTC Analytics AG, Zwingen, Switzerland). An electron impact (EI) source was used for the ionization of the molecules. The injector temperature was set at 280 °C and helium was used as carrier gas at a flow rate of 1 mL/min. The separation of the molecules was obtained by an HP-5MS capillary column (30 m, 0.250 mm i.d., 0.25 µm film thickness) from Agilent.

For EO quantitative analysis (measurement of carlina oxide content), the oven temperature was set at 60 °C, then increased to 120 °C at 7 °C/min, and then to 280 °C at 20 °C/min and maintained for 10 min, and finally increased to 300 °C at 20 °C/min and maintained for 1 min. The run time was 28.571 min. The transfer line was set at 280 °C and the temperature of the mass analyzer and the ionization source were set at 150 and 230 °C, respectively. The acquisition has been performed in SCAN mode (29–400 *m/z*). The EO was diluted (1:2000) in an HPLC-grade diethyl ether containing 200 µg/mL of undecane as an internal standard and 1 µL of this solution was injected in split mode (1:50). On the other hand, for EO qualitative analysis the oven temperature was set at 60 °C for 5 min, then increased to 220 °C at 4 °C/min, and then to 280 °C at 11 °C/min and maintained for 15 min, and finally to 300 °C at 15 °C/min and held for 0.5 min. The run time was about 67 min. The transfer line was set at 280 °C and the temperature of the mass analyzer and the ionization source were set at 150 and 230 °C, respectively. The acquisition has been carried out in SCAN mode (29–400 *m/z*). The EO samples were diluted using *n*-hexane in a 1:100 ratio and 1 µL injected in split mode (1:200).

Data computing was carried out using MSD ChemStation software (Agilent, Version G1701DA D.01.00) and the NIST Mass Spectral Search Program for the NIST/EPA/NIH EI and NIST Tandem Mass Spectral Library v. 2.3. The identification of carlina oxide was achieved by comparison with the standard (obtained as described in Section 4.6.3, whereas the other components by a combination of the temperature-programmed retention indices (RIs) and mass spectra (MS) overlapping with respect to those stored in FFNSC3, ADAMS, and NIST 17 libraries [21,22,23].

##### Linearity of the Quantification Method

The linearity was evaluated by injecting standard solutions at different concentrations of carlina oxide (100, 200, 300, 400, 600, 800, and 1000 µg/mL), containing 200 µg/mL of undecane as an internal standard. The calibration curve was constructed by plotting the carlina oxide peak areas against the response factor (RF), calculated as the ratio between the carlina oxide area and the undecane area. The linear regression equation obtained was *y* = 0.0054 *x* − 0.6766, while the calibration curve showed a coefficient of determination (R^2^) of 0.9939. All details about the calibration curve are reported in the Supplementary Materials (Section S3).

## 5. Conclusions

As a result of EOs being exploitable ingredients for agrochemical, pharmaceutical, nutraceutical, and cosmetic industries, it may be worthwhile to develop and improve new green extraction methods for the isolation of these products. In this study, a MAH process was optimized through a one-step statistical DoE, allowing the correlation of the characteristics of the obtained EOs with the conditions applied for the process using mathematical models. Despite the DoE approach being based on statistical analysis and prediction, in this study, complex data analysis, such as surface response methodology (RSM) and central composite design (CCD), were skipped. In fact, the results of the preliminary screening allowed us to optimize the process varying the ET only. Moreover, the results of the comparison between the HD and MAH techniques showed that MAH is more efficient than HD not only in terms of ET, but also in terms of EO yield (0.65 and 0.49% for MAH and HD, respectively, obtained in the same operative conditions). In conclusion, this approach might be useful for the implementation of the production of the *C. acaulis* EO, which is becoming popular in the agrochemical industry due to its highly promising insecticidal and acaricidal properties. Nonetheless, further experiments for the obtainment of this EO with MAH should be carried out, for example by varying some external parameters (e.g., cooling water temperature), to improve the protocol and hence, the final yield.

## Data Availability

Not applicable.

## Supplementary Materials

The following supporting information can be downloaded at https://www.mdpi.com/article/10.3390/plants12030622/s1**.** Section S1. Preliminary screening; Section S2. Parameters and results of extraction time study. Abbreviation for coded variables: MP (microwave power); ET (extraction time); W (percentage of water added to the seeds); Mo (moistening process); Mi (milling process); NA (not analysed); Section S3. (a) Calibration curve for GC-MS carlina oxide quantification, (b) GC-MS chromatogram of Carlina acaulis essential oil using undecane (as internal standard); Section S4. Images of milled (1.5 mm size particles) (left panel) and coarsely shredded (right panel) samples. The image of milled sample was acquired with a microscope (MT9000, Meiji Techno Co., Ltd., JP) equipped with a 3-megapixel CMOS camera (Invenio 3S, DeltaPix, DK) and objective lens of 10X. The image of coarsely shredded sample was acquired with a mobile phone camera equipped with CMOS 13-megapixel (4208 × 3120) sensor. Images were processed and calibrated with image pro-plus software.
