# Supplementary material for: Microwave-Assisted Hydrodistillation of the Insecticidal Essential Oil from Carlina acaulis: A Fractional Factorial Design Optimization Study
Source: Plants (Basel). 2023 Jan 31;12(3):622. doi: 10.3390/plants12030622 (PMC9921509; doi:10.3390/plants12030622)
Supplement: Supplementary file 1 [file plants-12-00622-s001.zip › plants-2182217-supplementary.docx]

Microwave-assisted hydrodistillation of the insecticidal essential oil from *Carlina acaulis*: a Fractional Factorial Design optimization study

Eleonora Spinozzi ^1^, Marta Ferrati ^1^, Desirèe Lo Giudice ^1^, Eugenio Felicioni ^1^, Riccardo Petrelli ^1^, Giovanni Benelli ^2^, Filippo Maggi ^1,^*, and Marco Cespi ^1^

^1^ Chemistry Interdisciplinary Project (ChIP) Research Center, School of Pharmacy, University of Camerino, Via Madonna delle Carceri 9/B, 62032 Camerino, Italy

^2^ Department of Agriculture, Food and Environment, University of Pisa, via del Borghetto 80, 856124 Pisa, Italy

***** Correspondence: filippo.maggi@unicam.it; Tel.: +39 0737 404506

Supplementary materials

## **Section S1 Preliminary screening.**

### **Section S1.1 Analysis of variance (ANOVA) output showing the effects of parameters affecting the *Carlina acaulis* essential oil extraction yield.**


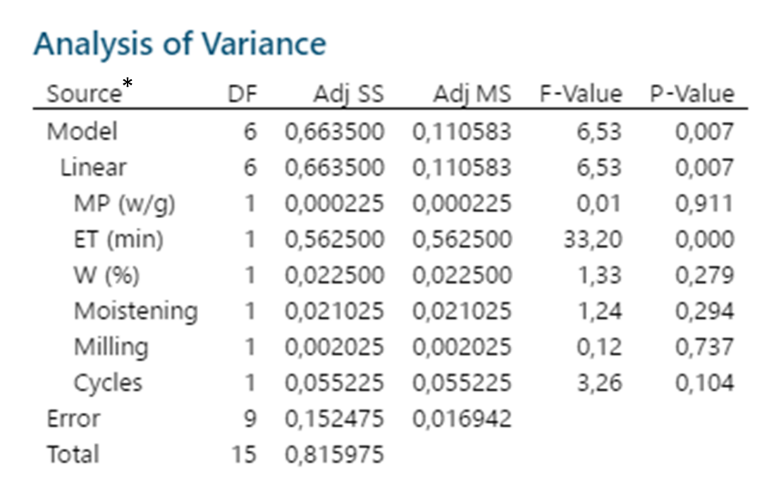


^*^Abbreviation for coded variables: MP (microwave power); ET (extraction time); W (percentage of water added to the seeds).**
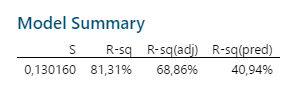
**


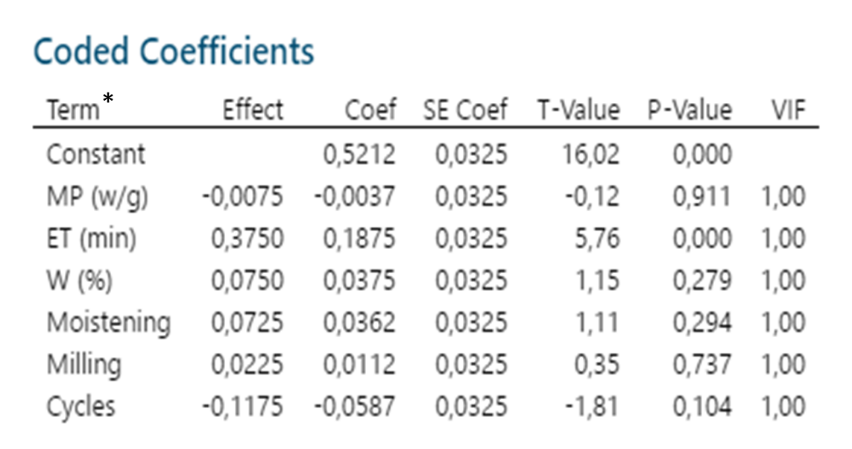


*Abbreviation for coded variables: MP (microwave power); ET (extraction time); W (percentage of water added to the seeds).

**
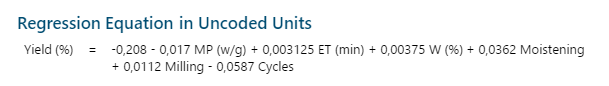
**

**
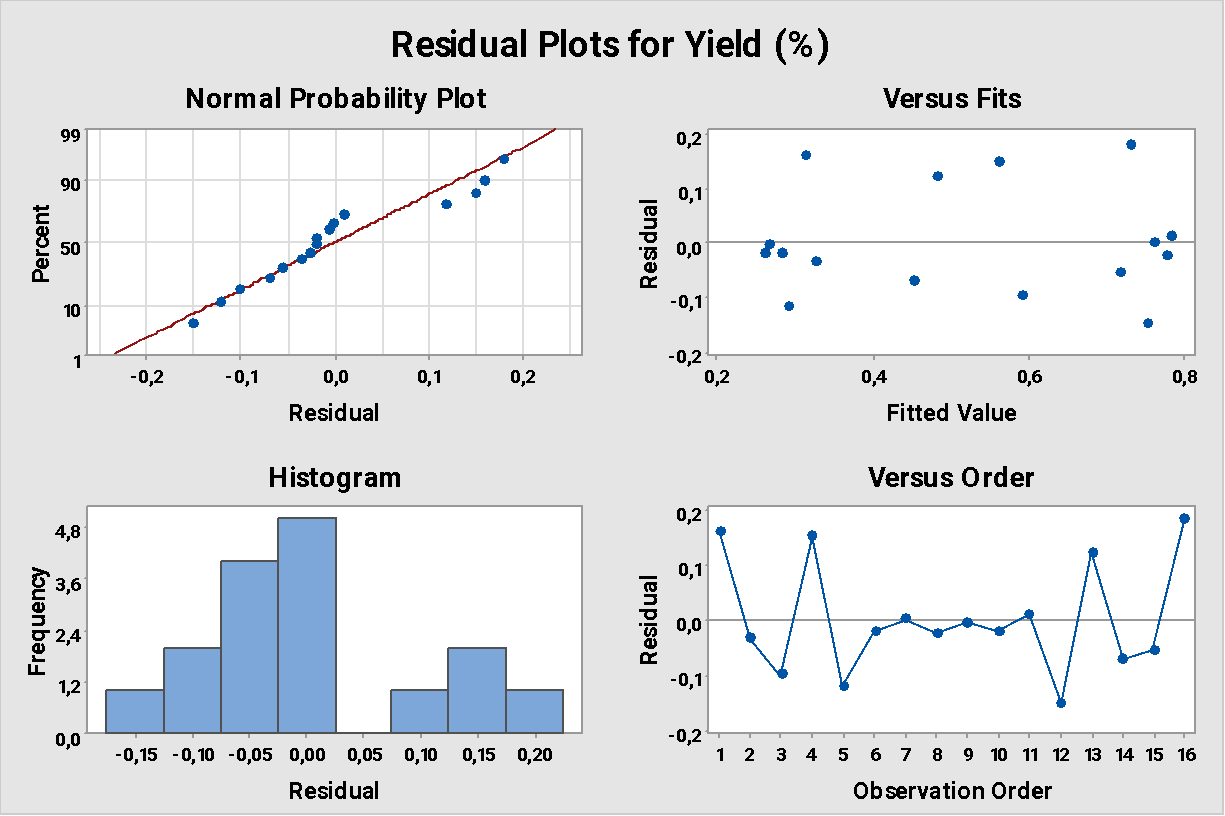
**

Abbreviation for coded variables: MP (microwave power); ET (extraction time); W (percentage of water added to the seeds).

### **Section S1.2 Analysis of variance (ANOVA) output showing the effects of parameters affecting the concentration of carlina oxide.**


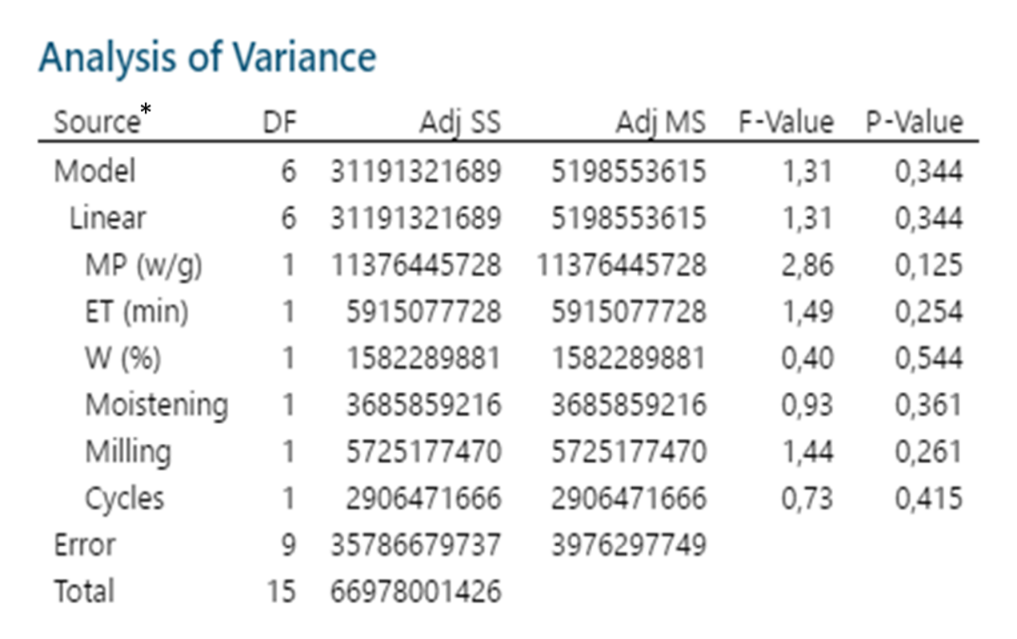


*Abbreviation for coded variables: MP (microwave power); ET (extraction time); W (percentage of water added to the seeds).


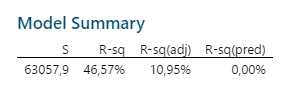


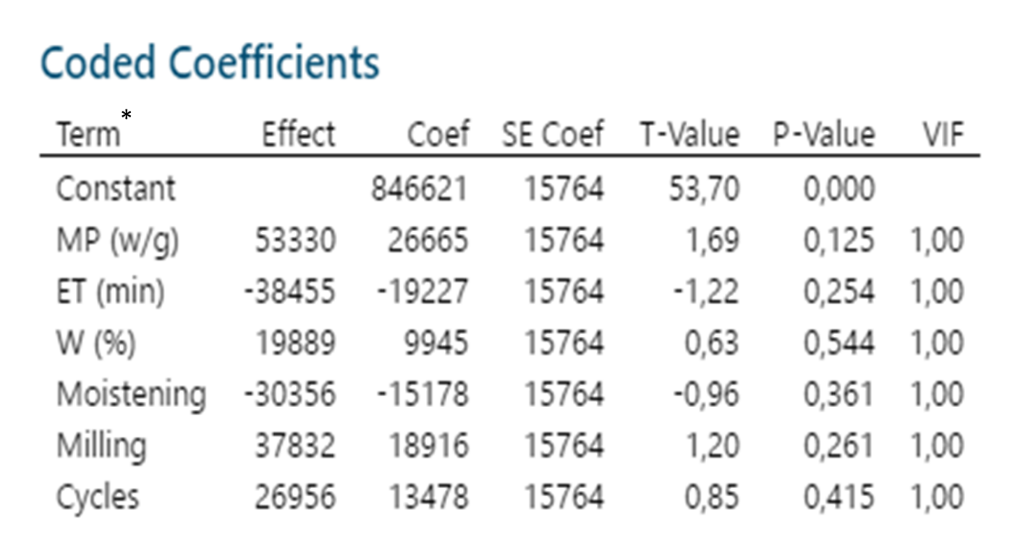


*Abbreviation for coded variables: MP (microwave power); ET (extraction time); W (percentage of water added to the seeds).
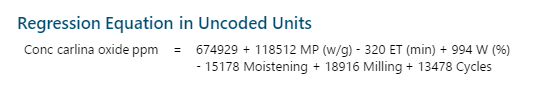


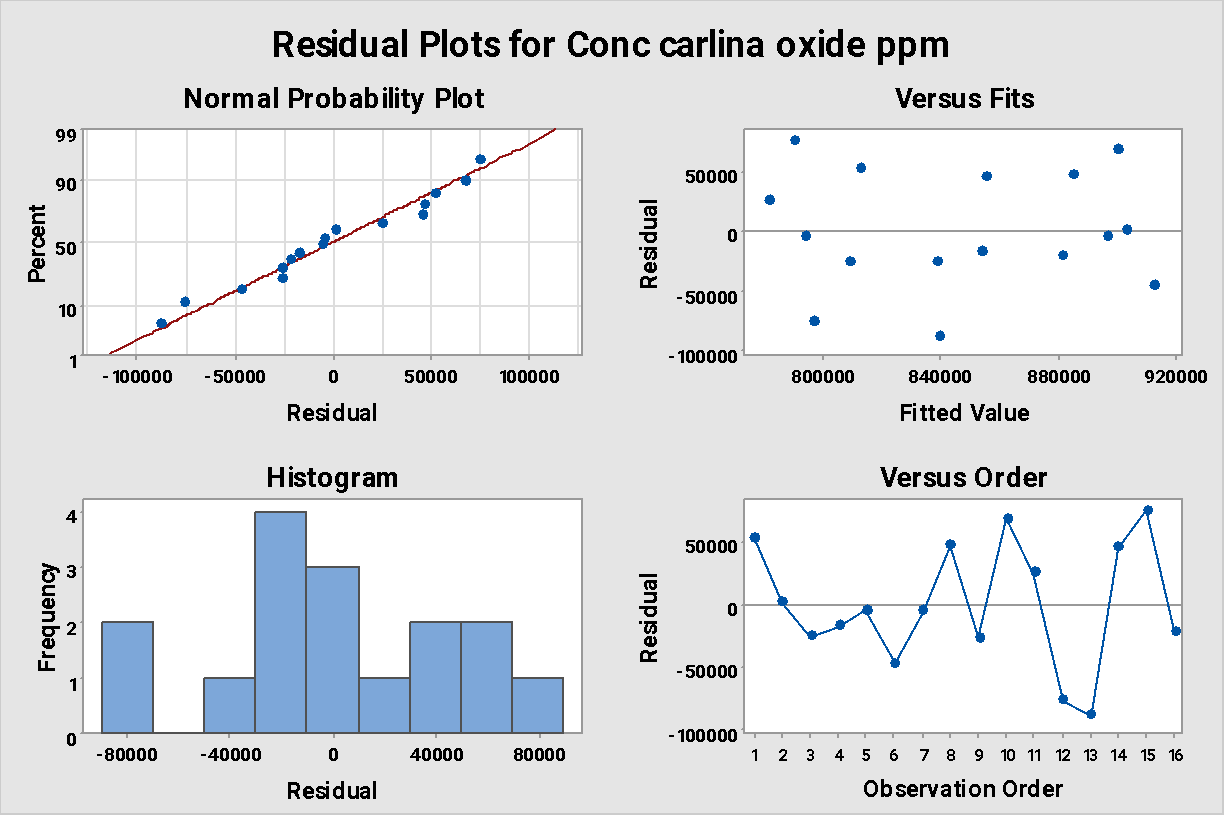


Abbreviation for coded variables: MP (microwave power); ET (extraction time); W (percentage of water added to the seeds).

## **Section S2 Effect of the extraction time.**

**Table S2.** Parameters and results of extraction time study. Abbreviation for coded variables: MP (microwave power); ET (extraction time); W (percentage of water added to the seeds); Mo (moistening process); Mi (milling process); NA (not analysed).

| MP (W) | ET (min) | Water (g) | Root (g) | Mi | Mo | Cycles | Yield (%) | Carlina oxide (µg/mL) |
| --- | --- | --- | --- | --- | --- | --- | --- | --- |
| 1000  1000  1000  1000  1000  1000  1000  1000 | 90  90  210  210  270  270  330  330 | 850  850  850  850  850  850  850  850 | 150  150  150  150  150  150  150  150 | NA  NA  NA  NA  NA  NA  NA  NA | NA  NA  NA  NA  NA  NA  NA  NA | NA  NA  NA  NA  NA  NA  NA  NA | 0.26  0.24  0.55  0.74  0.64  0.73  0.72  0.73 | 1.18E+06  9.32E+05  1.10E+06  8.88E+05  1.11E+06  1.01E+06  1.15E+06  1.00E+06 |

## **Section S3 (a) Calibration curve for GC-MS carlina oxide quantification.**


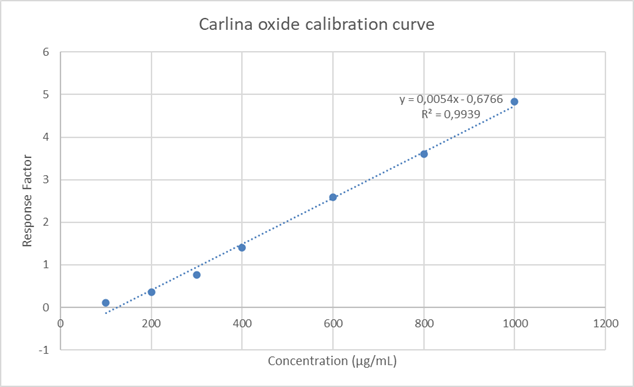


## **Section S3 (b) GC-MS chromatogram of *Carlina acaulis* essential oil using undecane (as internal standard)**


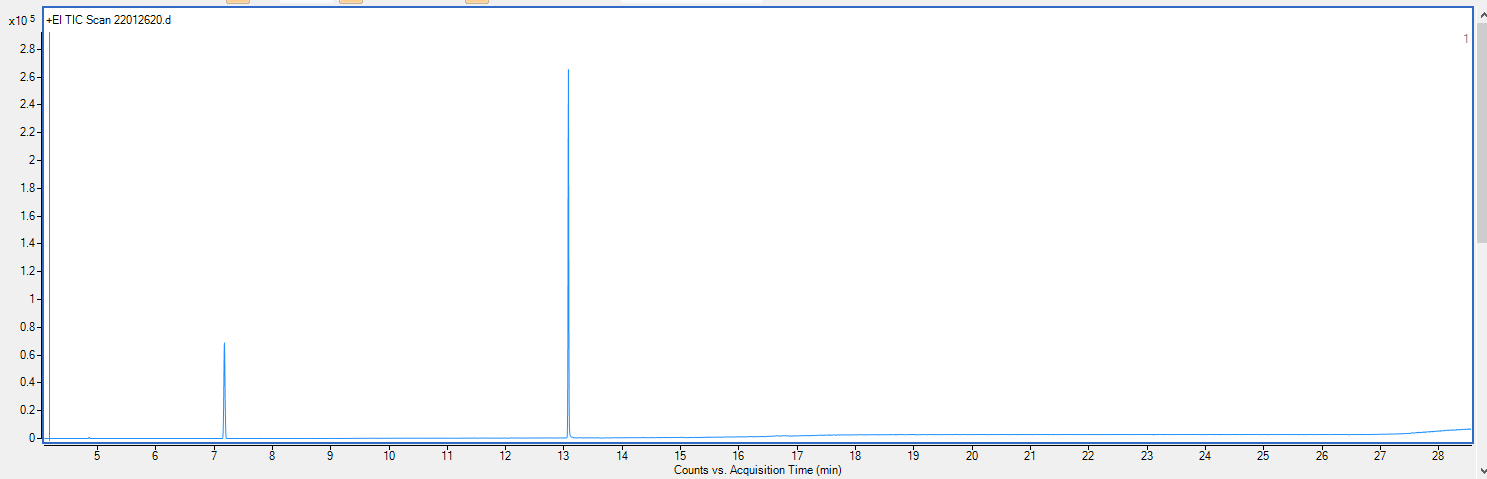


*n*-undecane

carlina oxide

## **Section S4 Effect of milling**


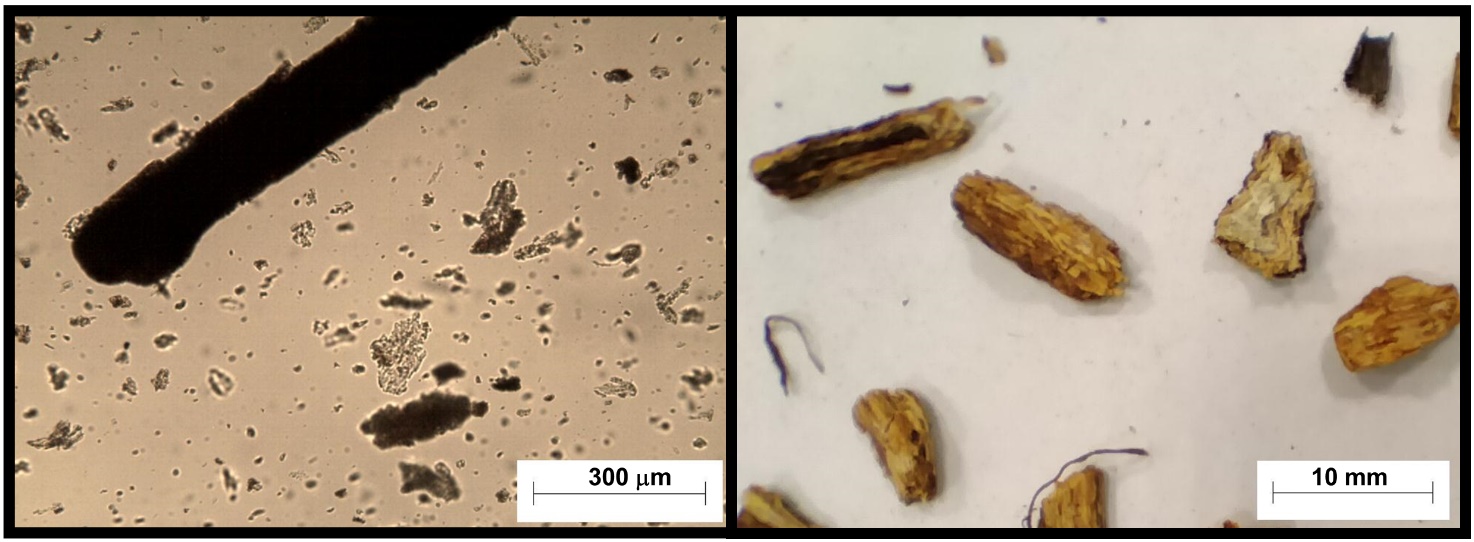


**Figure S4:** Images of milled (1.5 mm size particles) (left panel) and coarsely shredded (right panel) samples. The image of milled sample was acquired with a microscope (MT9000, Meiji Techno Co. Ltd, JP) equipped with a 3-megapixel CMOS camera (Invenio 3S, DeltaPix, DK) and objective lens of 10X. The image of coarsely shredded sample was acquired with a mobile phone camera equipped with CMOS 13-megapixel (4208x3120) sensor. Images were processed and calibrated with image pro-plus software.
